# A Highly Selective Analytical Method Based on Salt-Assisted Liquid-Liquid Extraction for Trace-Level Enrichment of Multiclass Pesticide Residues in Cow Milk for Quantitative Liquid Chromatographic Analysis

**DOI:** 10.1155/2023/1754956

**Published:** 2023-09-29

**Authors:** Habtamu Bekele, Weldegebriel Yohannes, Negussie Megersa

**Affiliations:** Department of Chemistry, College of Natural and Computational Sciences, Addis Ababa University, P.O. Box 1176, Addis Ababa, Ethiopia

## Abstract

In this study, a simple, inexpensive, selective, and fast salting-out assisted liquid-liquid extraction (SALLE) technique coupled with high-pressure liquid chromatography-diode array detection (HPLC-DAD) was developed for the extraction, preconcentration, and analysis of trace level seven multiclass pesticide residues in pasteurized and raw cow milk samples. The significant factors that affect the extent to which the target analytes are extracted, such as the type of extraction solvent and its volume, the type and concentration of salting-out salts, the pH of the solution, and the extraction time, have been investigated. Under optimum conditions, the correlation coefficient (*r*^2^) was obtained within a range of 0.9982–0.9997 for a broad linear range concentration of 2–1500 ng·mL^−1^. Reliable sensitivity was achieved with limits of detection (LODs) and limits of quantification (LOQs) ranging from 0.58–2.56 ng·mL^−1^ and 1.95–8.51 ng·mL^−1^, respectively. While precision with interday and intraday in terms of relative standard deviations (RSDs) was observed in the range of 1.97 – 7.88% and 4.52 – 8.04%, respectively. The results of the precision studies reveal that good repeatability and reproducibility (RSDs <9) were achieved, thus showing a low variability extraction of the developed method. Finally, the proposed and validated approach was effectively used to extract and determine pesticide residues in real milk matrices; however, the target analytes were not detected in all samples.

## 1. Introduction

Pesticides are chemical compounds used all over the world to control, prevent, or eliminate pests that threaten plants, animals, and human environments. It is well documented and known that their use increases agricultural productivity, though their residues greatly contaminate environmental components [1]. Nowadays, pesticide use has significantly increased throughout the world and similarly in Ethiopia [2] mainly as a result of the country's continuous agricultural reform. Out of the enormous quantities of pesticides applied, only less than 0.1% actually reach the intended pests; the remainder may end up on other environmental surfaces and accumulate in grasslands and feed additives given to cattle and other animals [3–5]. Pesticide residues at trace levels can be hazardous to unanticipated targets, posing a serious threat to human health and the ecosystem [2, 6, 7]. Humans come into contact with these chemicals through unsafe use, food, or the environment [5, 8]. Food security is a condition in which everyone, at all times, have physical, social, and economic access to sufficient quantities of wholesome foods to meet their dietary needs and food preferences for an active and healthy life [9]. Due to these facts, monitoring for pesticides in food matrices on a regular basis is crucial and has become one of the hot research topics these days [10, 11].

Pesticides typically exist in low concentrations in the environment and food matrices, and determination of these trace quantities requires various analytical instruments, including gas chromatography-mass spectrometry (GC-MS) [7, 10, 12], gas chromatography-tandem mass spectrometry (GC-MS/MS) [13], high-performance liquid chromatography (HPLC) combined with a diode array detector (DAD) [14–16], tandem mass spectrometry (MS/MS) [1, 5, 17–19], and ultraviolet detector (UVD) [20–22]. Most of these instruments provide good selectivity, sensitivity, low detection capacity, and so on; however, there are financial limitations to acquire them at laboratories that are not well equipped to meet the demanded requirements. However, relatively less expensive techniques, such as HPLC-DAD, are routinely used for monitoring of pesticide and other pollutant residues. In addition, when combined with a DAD detection system, HPLC procedures are typically favored over GC ones since HPLC is used without derivatization and is a sufficiently selective and sensitive analytical method [23]. Therefore, HPLC-DAD was chosen for monitoring of multiclass pesticides in milk samples for the designed sample preparation methods in the current study.

Dairy farming is one of the most profitable businesses in Ethiopia, particularly in the central Oromiya regional state. Furthermore, Ethiopia has one of the highest populations of cattle in Africa, estimated at 60 million heads, and around 90% of milk products are obtained from cows [24]. Milk is one of the required food item for mankind, but the question of its contamination with trace-level pesticides must be given attention, particularly when its handling personnel are untrained farmers and agricultural extension workers who lack knowledge of pesticide management, how to use for agronomy, and veterinary care are involved [8, 25]. Studies have revealed that despite the fact that most pesticides are often present in low concentrations, their persistence causes them to accumulate in animal tissues where they enter the food chain [5, 10, 11, 19]. Contamination of milk and milk products is extremely concerning because these foods hold a very special place in the diets of infants, young children, and the elderly for whom milk is a complete diet enriched with proteins, vitamins, fats, and essential minerals [26, 27].

The health concerns posed by trace pesticide residues in food can be significant, especially for young children whose enzymatic and metabolic systems are still developing [28–30]. Research on pesticide residues in the environment and various foods that have detrimental effects on human health is receiving special attention [30]. Because milk has dissolved proteins, carbohydrates, and minerals, it is difficult to recover trace-level multiclass pesticide residues with different physicochemical properties, and thus developing an amenable sample extraction technique and cleanup step is very crucial before chromatographic analysis [26].

Among numerous sample preparation techniques that have been performed to achieve efficient extraction of pesticides from milk and milk products, liquid-liquid extraction (LLE) [13, 31], solid-phase extraction (SPE) [16], dispersive solid-phase extraction (DSPE) [19], magnetic solid-phase extraction (MSPE) [28, 32], and solid-phase microextraction (SPME) [33, 34], Quick, easy, cheap, effective, rugged, and safe (QuEChERs) [14], pressurized liquid extraction (PLE) [12], and cloud point extraction [35, 36] were some of the reported works in the literature. The majority of these methods are labor intensive, time-consuming, and environmentally unfriendly, despite the fact that they offer clear advantages for extraction of pesticides from milk. Besides, as stated explicitly in published literature, industrially produced QuEChERs kits, SPME needles, and SPE cartilage materials are quite expensive [5, 6, 37].

Preconcentration of multiclass pesticide residues in food samples nowadays needs the development of analytical techniques that are miniaturized, efficient, simple, fast, and affordable. The most popular method, dispersive liquid-liquid microextraction (DLLME), is limited to the use of nonpolar, water-immiscible solvents with low dielectric constants and poor extraction efficiency of polar organic and inorganic compounds [38]. As a result, the introduction of salt-assisted liquid-liquid extraction (SALLE), an efficient extraction method for polar to moderately polar organic compounds, was made feasible by using more polar and water-miscible organic extraction solvents like acetonitrile, isopropanol, acetone, ethanol, and methanol, among others. In the SALLE method, organic solvent is separated from the mixture, and a two-phase system is created as a result of addition of inorganic salt [39]. When using inorganic or organic salts, the salting out effect increases the ionic strength of the solution and decreases the solubility of the weak electrolyte in water, which causes the target analyte to be extracted into the organic solvent, resulting in high extraction efficiency of polar or slightly polar target analyte in an aqueous sample [39]. The SALLE method produces extracts with solutes in organic solvent that may be evaporated and reconstituted with an appropriate solvent for preconcentration and analysis by HPLC or GC [40, 41]. On the other hand, in the SALLE methods, extraction solvents are compatible with the majority of analytical instruments, particularly chromatographic ones, making it possible to directly inject the extract into these methods of analysis [36, 42, 43].

SALLE has been used successfully to analyze pesticides in foods [20, 40, 44], biological matrices [36], and environmental water [45–47]. Researchers put a lot of work into making the method automated and high throughput during the development step to reduce processing time and chemicals required [35, 37]. Though various pretreatment technologies have been developed, the method of SALLE has still been widely used, since it integrates sample cleanup, preconcentration, and extraction in one single step and shares the advantages of the sample pretreatment technique gained from QuEChERS [48, 49]. Even though numerous advantages were reported, the application of SALLE for enrichment of multiclass pesticides in milk samples is scarce in the reported literature.

To the best of our knowledge, there are no reports in the literature on the use of the SALLE technique coupled with HPLC-DAD for simultaneous extraction and determination of multiclass pesticide residues including carbamate (carbrayl), organophosphate (methidathion), triazines (cyanazine, atrazine, and propazine), neonicotinoid (thiamethoxam), and strobilurin (azoxystrobin) in cow milk samples. Therefore, the present study was designed to develop, optimize, and validate a simple, fast, inexpensive, and an environment friendly (green) analytical technique based on SALLE, as an alternative for preconcentration and extraction of seven multiclass pesticide residues in cow milk samples.

## 2. Experimental

### 2.1. Chemicals and Reagents

The standards used in this study are of analytical reagent grade substances; methidathion was obtained from Sigma-Aldrich (St. Louis, MO, USA), and atrazine, cyanazine, and propazine were purchased from Dr. Ehrenstorfer GmbH (Augsburg, Germany). Azoxystrobin, carbrayl, and thiamethoxam were the products of Sigma-Aldrich (Steinheim, Germany). All the pesticide standards were of the highest purity, viz., >98%. Other common chemicals used in the study were also analytical-grade reagents while the solvents utilized including acetonitrile (ACN), dihexyl ether, ethyl acetate, and acetone acquired from Sigma-Aldrich (Steinheim, Germany), methanol (MeOH) received from Carlo Erba (Rodano, Italy), and iso-propanol (IPA) obtained from Sigma-Aldrich (Seelze, Germany) were HPLC-grade reagents. Magnesium sulphate anhydrous and ammonium sulphate were from Fine Chem Industries (Mumbai, India, 99%). Ammonium acetate (BDH Chemical Ltd, England, 96%) was obtained from VWR International (Radnor, PA, USA). Sodium sulphate and sodium acetate anhydrous were from (BDH Chemical Ltd, England, 96%). Common chemicals such as NaCl were obtained from Sigma-Aldrich (Steinheim, Germany), hydrochloric acid (HCl) was purchased from Sigma-Aldrich (St. Louis, MO, USA), and sodium hydroxide (NaOH) was the product of Merck chemicals (Darmstadt, Germany). Ultrapure water was prepared by purifying with a double distiller, a 8000 Aquatron water Still (Bibby Scientific, Staffordshire, UK), and a deionizer (EASY Pure LF, Dubuque).

### 2.2. Instruments and Equipment

Chromatographic analyses were carried out using the Agilent 1200 series HPLC system (Agilent Technologies, Waldbronn, Germany) outfitted with a quaternary pump, vacuum degasser, standard and preparative autosampler, thermostated column compartment, autosampler thermostat, and a diode array multiple wavelength detector. LC Chemstation software (B.02, 01-SR1) was used for sample processing and data acquisition. Chromatographic separation was performed using a ZORBAX ODS-C_18_ (150 × 3 mm, i.d., 3.5 *μ*m particle size) analytical column from Agilent Technologies. The sample solution pH was measured using an Adwa pH meter, model 1020, made by Adwa Hungary Kft. in Szeged, Hungary. For sample preparation, a centrifuge, Model 800, Jiangsu Zhenji insturuments Co., Ltd. (Jiangsu, China), a 15 mL centrifuge tube, Corning integrated (Corning, NY, Mexico), and an ultrasonic heater, Dacon®, were utilized.

### 2.3. Chromatographic Conditions

Chromatographic separations were achieved using the isocratic condition of a binary mobile phase, consisting of solvent A (40% ultrapure water) and solvent B (60% methanol). Prior to the sample injection, the HPLC column was equilibrated with the mobile phase for 10 min. Analysis was performed with a flow rate of 1 mL/min, column temperature of 35°C, injection volume of 15 *μ*L, and UV detection at 224 nm for all the target analytes. Peak area was used as instrumental response and comparison of the responses. Under these chromatographic conditions, baseline separation was maintained for all the target analytes.

### 2.4. Standard Solution Preparation

**Figure 1:**
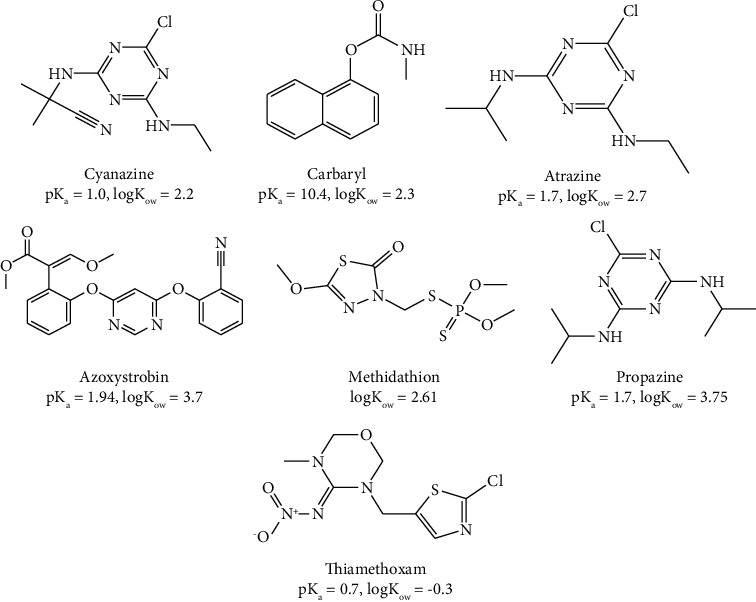
Chemical structure, common name, logK_ow_ (octanol-water partition coefficient), and pK_a_ (acid dissociation constant) of the multiclass pesticide compounds under study.

The stock standard solution of each target analyte, with the concentration of 0.1 mg/mL, was prepared by weighing the appropriate amount and dissolving it in methanol. Intermediate standard solutions of 10 *μ*g/mL were obtained by diluting the stock solution with ultrapure water. Other working solutions of lower concentrations were also prepared by diluting the intermediate solution in the ultrapure water. All standard solutions were stored in the refrigerator below 4°C, when not in use. The chemical structures, common names, abbreviations, the octanol-water partition coefficient (logP; at pH 7 and 20°C), and other relevant physicochemical properties of the target pesticides considered are shown in Figure 1.

### 2.5. Milk Samples

A total of 7 milk samples (one fresh raw milk collected from a dairy cattle farm and three pasteurized milk processed and packed by two dairy product suppliers) were taken. Pasteurized milk samples were bought from randomly selected local supermarkets in Addis Ababa, and raw milk samples were donated from a randomly selected dairy cattle farm in Sheger city (in sululta subcity) in April 2023 for the multiclass pesticide residue analysis. After arrival at the laboratory, the pasteurized milk samples in their original packing and raw milk in a brown bottle were kept in a refrigerator at 4°C until the time of analysis, when not in use. Note that the names of the producers have been kept confidential to protect their business and reputation.

### 2.6. Procedure of SALLE

Aliquots of 0.5 mL of milk sample were placed in a 15 mL falcon centrifuge conical bottom tube and then diluted to 5.0 mL with ultrapure water (pH 8.0) to reduce the matrix effect of the sample. The sample solution pH was adjusted using 0.1 M HCl or 0.1 M NaOH solution and spiked with appropriate amount of mixed standard solutions of the pesticides. The sample solution was then kept to stand for 20 min to equilibrate, and 1.0 mL ACN was added and vortexed for 0.5 min. This was followed by the addition of 2.0 g MgSO_4_ to the mixture and vortexed for an additional 2 min to dissolve the salt to be used as a salting out agent. After centrifuging the resulting content at 4000 rpm for 5 min, 500 *μ*L of the supernatant was carefully withdrawn with a micropipette and transferred to a vial filtering through a 0.22 *μ*L filter membrane. Then, 15.0 *μ*L was injected into the HPLC–DAD system for extract analysis.

### 2.7. Statistical Analysis

Descriptive statistical analysis of means, standard deviations, and relative standard deviations for data obtained during parameter optimizations and validations of the method was performed using Microsoft Office Excel 2010 software, and figures were drawn using Origin 2019b software.

## 3. Results and Discussion

### 3.1. Optimization of the SALLE Procedure

This research work was designed with the interest of developing an efficient analytical methodology which is miniaturized, simple, fast, and cost-effective for the analysis of multiclass pesticide residues. Attainment of the desired efficiency was achieved by making use of a single sample preparation process to be able to analyze seven multiclass pesticide residues simultaneously. During method development, experiments were conducted to optimize different extraction parameters including the type and volume of the organic solvent, type and amount of salt, pH of the sample solution, and vortex time. These experimental conditions were evaluated by spiking reagent water at concentrations of 100 *μ*g/L for CAR; 130 *μ*g/L for THE, CYZ, ATZ, and PRZ; 260 *μ*g/L for AZO; and 390 *μ*g/L for MET. All the experiments were performed in triplicate (experimental) and doublet reading (instrumental). The mean peak area studies that may have impacts on the SALLE extraction efficiency were taken as instrument response when establishing the optimum experimental conditions for the following parameter under study.

#### 3.1.1. Selection of Extraction Organic Solvent

**Figure 2:**
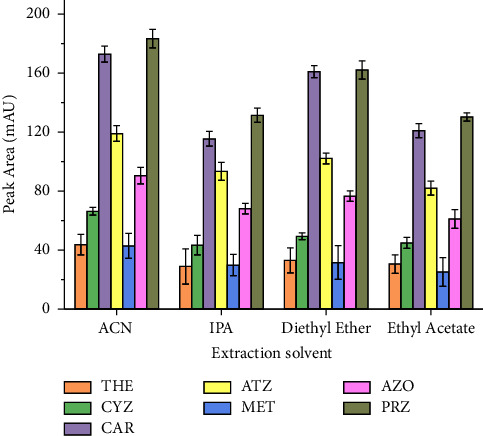
Effect of extraction solvent type. Extraction conditions: sample size, 5 mL; amount of salt added, 30% NaCl (m/v); pH of solution, 7.0; vortex agitation time, 1 min; volume of each extraction solvent, 2 mL; centrifugation speed, 4000 rpm for 5 min; *n* = 6.

Selection of an appropriate extraction solvent is the critical step in a SALLE procedure. The organic solvents with the desired characteristics such as high capability to dissolve the analyte, miscibility with water, ease of inducing phase separation upon addition of the appropriate salt and having good chromatographic behavior were tested as extraction solvent. Moreover, the solvent peak should not interfere with the analyte peak under the selected HPLC conditions. In this work, solvents such as MeOH, ACN, IPA, acetone, diethyl ether, and ethyl acetate were tested. A series of experiments were performed using a 5 mL ultrapure water sample containing 30% NaCl (m/v) and 2 mL of each organic solvent with the exception of methanol and acetone, in which the two phase systems were not observed. Similar observations were also noted for methanol and acetone and reported in literature by other workers [20, 22, 50]. The reason for the absence of phase separation in methanol could be due to the high polarity of methanol caused by its hydroxyl group and the hydrogen bond formed between this solvent and water which as a result increases its solubility [51].  Figure 2 depicts the observed maximum peak area when ACN was used as the extraction solvent. This might be attributed to its closer polarity with water and its promising protein precipitation reagent for milk [52]. Additional advantages of using ACN as an extraction solvent are its ability to extract a wide range of compounds [53] caused by its higher polarity and its less toxic and less harmful nature compared to other common extraction solvents. These characteristics also make it more suitable from the viewpoint of green chemistry. Thus, ACN is selected to be used as extraction solvent in this study.

#### 3.1.2. Volume of the Extraction Solvent Effect

**Figure 3:**
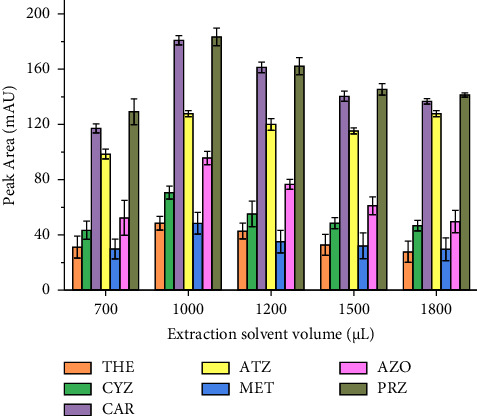
Effect of extraction solvent volume. Extraction conditions: sample size, 5 mL; extraction solvent, acetonitrile; amount of salt added, 30% NaCl (m/v); pH of solution, 7.0; vortex agitation time, 1 min; centrifugation speed, 4000 rpm for 5 min; *n* = 6.

The volume of the extraction solvent is a very crucial parameter that influences the extraction performance of the SALLE technique since it affects the amount of analyte solubility in the sample solution [51]. Generally, the volume of extraction solvent used should be as low as possible to achieve the highest possible enrichment and the least toxicity hazards for environment. In this context, the influence of the ACN volume on the extraction efficiency was investigated between 700 and 1800 *μ*L. As shown in Figure 3, peak areas of all the analytes increased with the volume of ACN from 700 to 1000 *μ*L and then decreased upon further increase in the volume of the ACN. With low volumes, i.e., lower than 700 *μ*L, the interface between the extraction solvent and the aqueous phases was not clear, and collection of the organic layer was found to be difficult. A decrease in extraction efficiency above 1000 *μ*L may be due to the dilution effect resulting from the higher volume of the organic phase obtained after extraction, and hence further higher volumes were not performed [22]. Hence, based on the observed experimental results, 1000 *μ*L ACN was selected as the optimum volume in all the subsequent experiments.

#### 3.1.3. Effects of the Salt Type

**Figure 4:**
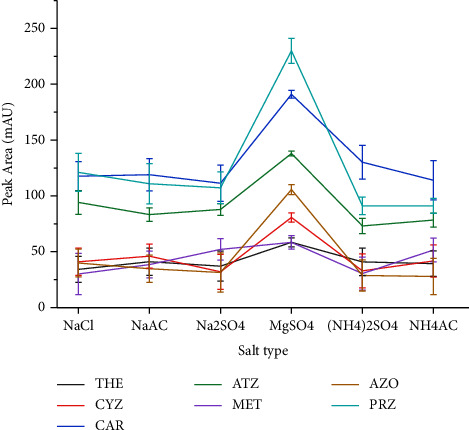
Effect of the salt type. Extraction conditions: sample size, 5 mL; extraction solvent, acetonitrile; extraction solvent volume; 1000 *μ*L; amount of each salt added, 30% (m/v); pH of solution, 7.0; vortex agitation time, 1 min; centrifugation speed, 4000 rpm for 5 min; *n* = 6.

The solubility of both the analytes as well as the extraction solvent in the aqueous phase could be decreased by salt addition, and this in turn enhances the analytes transfer into the organic phase [44, 48]. As different salts have the capacity to cause different degrees of phase separation [52], in this study, the effect was evaluated by addition of the salts such as NaCl, (Na)_2_SO_4_, MgSO_4_, (NH_4_)_2_SO_4_, and **NH_4_CH_3_CO_2_**, using 30% (m/v) of each salt, as potential salting out agent. It was observed that all salts could induce phase separation, but as it can be seen from Figure 4, the highest instrumental response for all analytes was obtained when MgSO_4_ was used as the salting out agent. This could be due to its high ionic strength per unit concentration in the aqueous phase [43].

#### 3.1.4. Effect of Salt Concentration

**Figure 5:**
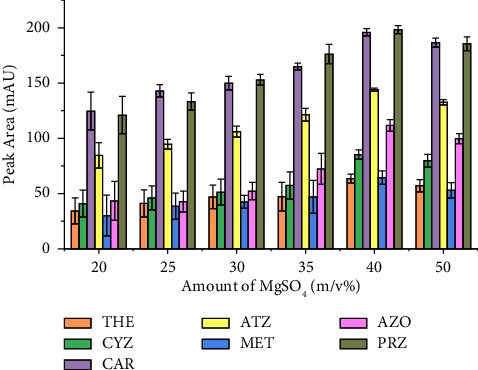
Effect of the amount of MgSO_4_. Extraction conditions: sample size, 5 mL; extraction solvent, acetonitrile; extraction solvent volume, 1000 *μ*L; salt type, MgSO_4_; pH of solution, 7.0; vortex agitation time, 1 min; centrifugation speed, 4000 rpm for 5 min, *n* = 6.

Varying salt concentrations may cause the degrees of phase separation to vary [43, 54]. A salting-out study was carried out by adding different amounts of MgSO_4_ in the range of 0.75 g–2.5 g (or 15–50%, m/v) in the aqueous sample solution. It was shown that in Figure 5, the peak area of the target analytes was slightly increased as the concentration of the salt increases from 1 g to 2 g. However, at higher concentrations, the peaks were observed to slightly decrease for all the target analytes, and thus 40% m/v (2 g) was chosen to be the optimum for the following experiments. Similar quantities of this salt were found to cause a significant salting-out effect in the SALLE analytical method, reported in the literature and employed for fruit juice, yogurt, and carbonated drink matrices [41, 44].

#### 3.1.5. Effect of Sample pH

**Figure 6:**
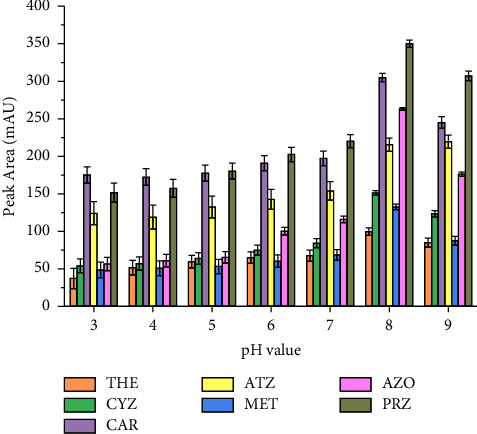
Effect of pH. Extraction conditions: sample size, 5 mL; extraction solvent, acetonitrile; extraction solvent volume, 1000 *μ*L; salt type, MgSO_4_; amount of MgSO_4_ added, 40% (m/v); vortex agitation time, 1 min; centrifugation speed, 4000 rpm for 5 min; *n* = 6.

In SALLE, the sample solution pH also has a significant role on the extraction efficiency of the multiclass pesticides, as it affects the extent of their ionization as well as the solubility in aqueous media [35, 51, 55]. The effect of this parameter was evaluated by carrying out a series of experiments varying the pH values from 3.0 to 9.0 in the aqueous solution. These pH values were adjusted using HCl and NaOH. The experimental results obtained revealed that pH 8 was the optimum value, as shown in Figure 6. This could mainly be associated with the enhanced stability of the target analytes in the weakly alkaline solution, while they were easily degraded in acidic and strongly alkaline environments [4]. Therefore, pH 8 was selected as the optimum value for the subsequent studies.

#### 3.1.6. Effect of Centrifugation Time

In SALLE procedures, optimizing the time required for phase separation is also an important analytical step, in order to obtain a clear extract [44]. In order to establish the optimum conditions: centrifugation time was varied between 1 and 7 min, with a 2 min interval, at a constant speed of 4000 rpm. Based on the peak areas representing the target analytes, the highest results were obtained at the centrifugation time of 5 min (shown in supplementary material Figure S1). Therefore, centrifugation time of 5 min was selected as the optimum time for the subsequent studies.

#### 3.1.7. Effect of Vortex Agitation Time

**Figure 7:**
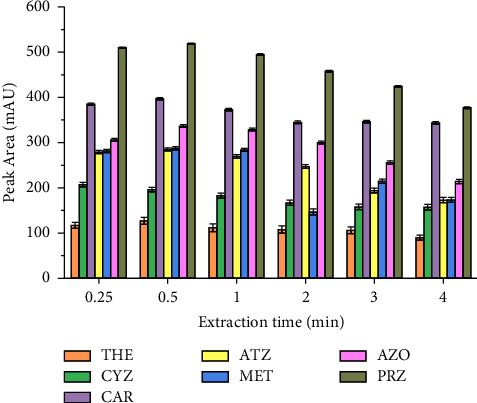
Effect of the extraction time. Extraction conditions: sample size, 5 mL; extraction solvent, acetonitrile; extraction solvent volume, 1000 *μ*L; salt type, MgSO_4_; amount of MgSO_4_ added, 40% (m/v); pH of solution, 8.0; centrifugation speed, 4000 rpm for 5 min; *n* = 6.

Mass transfer is a time-dependent process and one of the most important factors in most of the extraction procedures [44]. Vortex was performed to strengthen the contact between acetonitrile and the aqueous sample solution (i.e., influence the kinetics of the extraction), thus facilitating the formation of the two-phase system. Besides, in the present study, vortex agitation was also employed to enhance the dissolution of the salting-out salt. Therefore, a vortex time was evaluated in the range of 0.25–4 min, at the maximum vortex speed, and thus a slight increase of peaks was obtained when the vortex time increased from 15 sec to 30 sec. This indicates that the diffusion of pesticides from the sample to the acetonitrile medium was found to require a short time. A decrease in extraction efficiency after 30 sec (Figure 7) may be associated to the back extraction. Thus, extraction time of 30 sec was chosen in the present study.

### 3.2. Analytical Performance of the Proposed Method

#### 3.2.1. Calibration Curves and Precision Study

The proposed analytical method was validated through linearity and analytical figures of merit under optimal conditions. Linearity validation of the method was performed with the establishment of the linear calibration using external standard, and the corresponding curves (supplementary information, Figure S2) were generated by plotting the area of the analyte peak against the standard concentration (ng/mL). Good linearity, with a correlation coefficient >0.998 was obtained for all the target analytes considered in this study over the studied concentration range (Table 1). Individual chromatograms of the target analyte considered in this study are given in Figure S3.

The precision of the proposed method was also evaluated in terms of repeatability (intraday precision) and reproducibility (interday precision). To study repeatability of the method, pasteurized milk sample one (PSM) was spiked with the mixture of seven pesticides at two concentration levels (*μ*g L^−1^): Level 1 : 10 for CAR; 15 for THE, CYZ, ATZ, and PRZ; 30 for AZO; and 45 for MET, and Level 2 : 100 for CAR; 130 for THE, CYZ, ATZ, and PRZ; 260 for AZO; and 390 for MET. The sample was extracted in triplicate and injected in duplicate on the same day under the optimized experimental conditions. The reproducibility of the method was also validated using the same milk sample at concentration values used above to evaluate reproducibility for four consecutive days, following single extraction and injection. As shown in Table 1, the RSD % of the method was in the range of 1.97–7.88 for intraday and 4.52–8.04 for interday. The results of the precision studies reveal that good repeatability and reproducibility (RSD <9) were achieved, thus showing a low variability extraction technique [35, 56].

#### 3.2.2. Sensitivity

The sensitivity of the method guaranteed the detection and confirmation of pesticide residues in milk found at levels below or above the limits of detection (LODs). The calculations for LODs and limits of quantitation (LOQs) were based on the standard deviation (*σ*) of the seven extraction responses of blank milk for each type of milk samples and the slope of the calibration curve (S) using equations 3 × *σ*/*S* and 10 × *σ*/*S*, respectively [55]. The results are given in Table 1, showing that the LODs ranged from 0.58 to 2.56 ng·mL^−1^ while LOQs from 1.95 to 8.51 ng·mL^−1^.

### 3.3. Applications of the SALLE Method to Real Milk Samples

The suggested method's accuracy was validated using three real milk samples including pasteurized milk sample one (PMM), pasteurized milk sample two (PSM), and raw sululta milk sample (RSM). None of the tested milk samples produced signals corresponding to values above the LODs when the unspiked sample was evaluated to determine whether the seven selected target analytes were identified or not. The observed results may indicate that the samples tested were either free of pesticide residues or contained amounts below the detected limits. The average relative recovery (RR%) of each sample spiked at two concentration levels and extracted in triplicate was used to determine the accuracy of the proposed SALLE technique (Table 2). Relative recovery was intended using the standard addition on the blank real samples to evaluate the methods accuracy [21, 22, 35]. Mean relative recoveries (RR %) at two concentration levels were in the range of 85.9–108.8%, with %RSD <11.5 for the studied milk samples. The results obtained for recovery were in the acceptable range [56], indicating that the matrices of milk samples have no intense effect on the performance of the proposed method. Similar results were also reported by other workers both for accuracy and precision for the analysis of pesticides in the studied milk [35].

**Figure 8:**
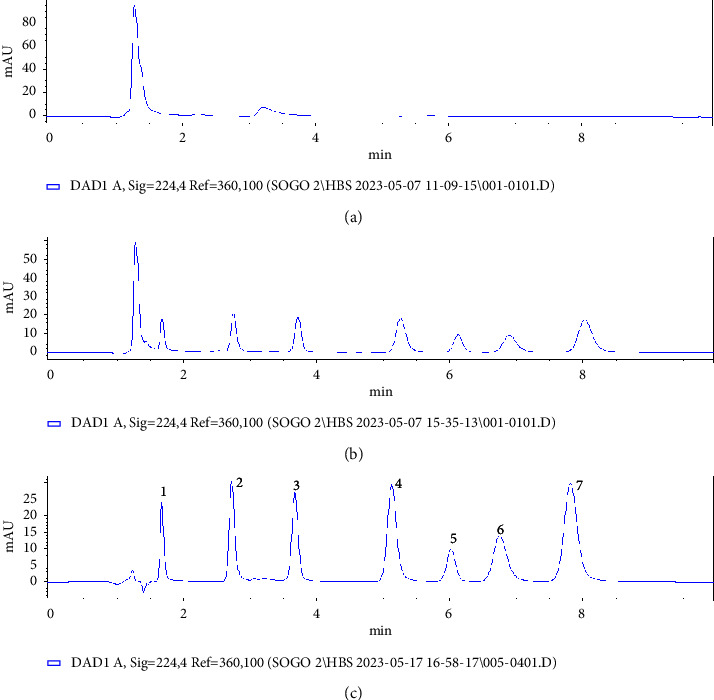
Typical chromatograms of blank (a), unspiked (b), and spiked (c) PSM milk samples at a concentration level 2 (100 *μ*g/L for CAR; 130 *μ*g/L for THE, CYZ, ATZ, and PRZ; 260 *μ*g/L for AZO; 390 *μ*g/L for MET). Extraction conditions: sample size, 5 mL; extraction solvent, acetonitrile; extraction solvent volume, 1000 *μ*L; salt type, MgSO_4_; amount of MgSO_4_ added, 40% (m/v); extraction time, 0.5 min; pH of solution, 8.0; centrifugation speed, 4000 rpm for 5 min. Peaks identifications: 1, thiamethoxam; 2, cyanazine; 3, carbrayl; 4, atrazine; 5, methidathion; 6, azoxystrobin; 7, propazine.

The chromatograms of the target multiclass pesticide residues in the PSM milk sample before and after spiking at concentration (level 2) used for precision study using the developed SALLE methods are shown in Figure 8. The separation of target analytes in the chromatogram obtained using reversed-phase high-performance liquid chromatography is in the order of their polarity in which the more polar elute first and the less polar one retained more (Figure 1, logK_ow_ value). The chromatograms selectivity was assessed by comparing blank and fortified sample peaks. It is evident from these chromatograms that absence of the chromatographic peaks from coextracted components and is well resolved for all analytes, demonstrating a high level of selectivity at the same retention time as the target pesticides. Therefore, the reported chromatogram endorses the selectivity of the developed SALLE technique. The other milk samples evaluated by this study also had the same profiles (supplementary information, Figures S4 and S5).

### 3.4. Comparison of the Proposed Method to Other Previously Reported Methods for the Analysis of Milk Samples

The presented analytical method, i.e., SALLE-HPLC-DAD for preconcentration and determination of multiclass pesticide residues was compared with other methods reported in the literature, such as dispersive liquid-liquid microextraction with gas chromatography mass spectrometry (DLLME-GC-MS) [17], solid-phase extraction with high performance liquid chromatography coupled with ultraviolet detector (SPE-HPLC-UV) [16], cloud point extraction with HPLC-UV (CPE-HPLC-UV) [3], head space solid-phasemicroextraction with GC-MS (HS-SPME-GC-MS) [33], quick, easy, cheap, effective, rugged, and safe (QuEChERS) coupled with HPLC and diode array detector (QuEChERS-HPLC-DAD) [14], and dispersive solid-phase extraction combined DLLME with HPLC-DAD (DSPE-DLLME-HPLC-DAD) [57], and the results are shown in Table 3. As can be seen, in terms of the LODs, precisions and accuracy of the present method were better than or comparable to those of the other methods applied for extraction of pesticides from the same type of matrices, i.e., milk sample. For HS-SPME [33] and SPE [16] methods, there may be the problem of facing sample carryover effects which leads to false-positive results. The proposed SALLE is simple, and unlike the SPE method, it does not require multisteps conditioning, washing, loading, and elution [5, 6]. In addition, the proposed method is found to use simpler equipment and exhibits a wider linear range, integrated pretreatment and preconcentration in a single step, which would make the procedure simpler, cost-effective, time saving, and eco-friendly.

## 4. Conclusions

In this study, the SALLE-HPLC-DAD analytical technique, that is, simple, fast, and green, was developed and optimized for routine monitoring and quantitative determination of seven multiclass pesticide residues with a wide range of physicochemical properties including methidathion, atrazine, azoxystrobin, cyanazine, carbrayl, thiamethoxam, and propazine in samples of raw and pasteurized milk. Compared with more traditional extraction techniques like LLE and SPE, this method uses a significantly smaller extraction solvent and sample volume. For all of the experimental factors taken into account during the investigation, the approach was optimized utilizing univariate methods. The optimized method offers sufficient accuracy, precision, linearity, and sensitivity under optimum extraction conditions in a short extraction time. No matrix interferences were coextracted or seen in the analysis at their respective retention times while this method was being used to extract trace-level pesticides from milk samples. Comparatively to other reported research that used hazardous halogenated organic solvents as extraction solvents, the extraction solvents used in the current extraction approach are more environmentally benign. As a result, the trace level enrichment of multiclass pesticides using the SALLE analytical technique could be thought of as a good alternative for selective and sensitive extraction and practical assessment of multiclass pesticide residues in milk as well as enrichment of other trace compounds in complex samples in routine laboratory analysis.

## Figures and Tables

**Table 1:** Analytical figures of merit for the SALLE technique combined with HPLC-DAD for multiclass pesticide residues under study.

Analyte	Linear range (ng/mL)	Regression equation	LOD (ng/mL)	LOQ (ng/mL)	*r* ^2^	^a^Repeatability (RSD %, *n* = 6)		^a^Reproducibility (RSD%, *n* = 12)	
THE	10–750	*y* = 0.116*x* + 2.3401	2.27	7.58	0.9993	^b^2.02	^c^3.38	^b^5.56	^c^7.77
CYZ	3–1000	*y* = 0.1776*x* + 2.7101	1.43	4.76	0.9997	3.54	2.05	6.57	4.52
CAR	2–750	*y* = 0.3345*x* + 2.8145	1.76	5.88	0.9992	5.50	5.13	7.32	5.13
ATZ	10–1000	*y* = 0.2883*x* + 6.8847	0.68	2.26	0.9994	3.11	1.97	8.13	6.57
MET	8–1500	*y* = 0.0384*x* + 1.8033	2.56	8.51	0.9991	4.40	4.04	7.52	6.65
AZO	5–1500	*y* = 0.1033*x* + 2.7038	2.05	6.84	0.9991	6.44	7.88	5.99	8.04
PRZ	3–1000	*y* = 0.4143*x* + 8.486	0.58	1.95	0.9982	2.88	5.13	7.67	7.59

^a^Validated using a pasteurized milk sample one (PSM) sample. ^b^Level 1 : 10 *μ*g/L for CAR; 15 *μ*g/L for THE, CYZ, ATZ, and PRZ; 30 *μ*g/L for AZO; 45 *μ*g/L for MET. ^c^Level 2 : 100 *μ*g/L for CAR; 130 *μ*g/L for THE, CYZ, ATZ, and PRZ; 260 *μ*g/L for AZO; 390 *μ*g/L for MET.

**Table 2:** Relative recovery (RR) values of the proposed method in the milk samples.

Sample	Spiked level	Analytes						
		THE	CYZ	CAR	ATZ	MET	AZO	PRZ
		%RR (%RSD, *n* = 3)						
PSM	Level 1	95.7 (2.6)	93.0 (4.9)	92.8 (4.3)	87.7 (7.3)	90.2 (5.5)	92.8 (6.1)	108.8 (3.3)
	Level 2	93.3 (4.2)	94.2 (3.0)	104.6 (5.5)	92.3 (8.0)	87.5 (4.8)	89.7 (5.4)	96.7 (2.7)

PMM	Level 1	97.9 (7.3)	93.2 (4.6)	91.7 (4.6)	87.9 (7.0)	91.6 (4.1)	85.9 (4.6)	108.3 (8.9)
	Level 2	92.9 (7.0)	94.3 (3.1)	97.5 (5.0)	91.3 (3.4)	90.0 (4.0)	88.5 (7.0)	92.9 (3.3)

RSM	Level 1	96.0 (7.8)	86.3 (8.9)	88.4 (11.4)	88.2 (8.4)	85.3 (10.3)	88.6 (5.2)	94.2 (11.3)
	Level 2	97.1 (10.6)	91.6 (6.2)	97.0 (8.0)	92.5 (5.1)	92.8 (9.2)	87.1 (6.2)	89.5 (10.9)

PSM, pasteurized milk sample one; PMM, pasteurized milk sample two; and RSM, raw sululta milk sample. Level 1 : 10 *μ*g/L for CAR; 15 *μ*g/L for THE, CYZ, ATZ, and PRZ; 30 *μ*g/L for AZO; 45 *μ*g/L for MET. Level 2 : 100 *μ*g/L for CAR; 130 *μ*g/L for THE, CYZ, ATZ, and PRZ; 260 *μ*g/L for AZO; 390 *μ*g/L for MET.

**Table 3:** Comparison of the proposed method with other methods applied for the extraction and determination of pesticides in milk samples.

Methods	Detection	Extraction time (min)	LR (*μ*g L^−1^)	LOD (*μ*g L^−1^)	RSD	RR	Ref.
CPE	HPLC–UV	30	50–2000	6.79–11.2	1.41–5.99	70.5–96.9	[3]
QuEChERS	HPLC–DAD	1	—	20–60	1–23	35–131	[14]
SPE	HPLC–UV	20	1–320	0.12–0.40	5.1–6.3	86–110	[16]
DLLME	GC–MS	0.5	2–1000	0.9–5.0	1.02–4.18	86.15–112.45	[17]
HS-SPME	GC–MS	45	6.5–56	2.2–10.9	6.1–29.5	—	[33]
DSPE–DLLME	HPLC–DAD	11	0.57–1000	0.17–0.36	3.3–7.2	79–92	[56]
SALLE	HPLC–DAD	0.5	2−1500	0.58–2.56	1.97–8.04	85.9–108.8	This work

“—”, not reported.

## Data Availability

The data that support the findings of this study are available from the corresponding author upon reasonable request.

## Supplementary Materials

Supplementary Materials Figure S1: effect of centrifugation time. Extraction conditions: sample size, 5 mL; extraction solvent, acetonitrile; extraction solvent volume, 1000 *μ*L; salt type, MgSO_4_; amount of MgSO_4_ added, 40% (m/v); extraction time, 0.5 min; pH of solution, 8.0; and centrifugation speed, 4000 rpm; *n* = 6. Figure S2: calibration curve of (a) thiamethoxam, (b) cyanazine, (c) carbrayl, (d) atrazine, (e) methidathion, (f) azoxystrobin, and (g) propazine. Figure S3: chromatograms of individual pesticides: (1) thiamethoxam; (2) cyanazine; (3) carbrayl; (4) atrazine; (5) methidathion; (6) azoxystrobin; and (7) propazine. Figure S4: typical chromatograms of blank (A), unspiked (B), and spiked (C) PMM milk sample at concentration level 2 (100 *μ*g/L for CAR; 130 *μ*g/L for THE, CYZ, ATZ, and PRZ; 260 *μ*g/L for AZO; and 390 *μ*g/L for MET). Extraction conditions: sample size, 5 mL; extraction solvent, acetonitrile; extraction solvent volume, 1000 *μ*L; salt type, MgSO_4_; amount of MgSO_4_ added, 40% (m/v); extraction time, 0.5 min; pH of solution, 8.0; and centrifugation speed, 4000 rpm for 5 min. Peaks identifications: (1) thiamethoxam; (2) cyanazine; (3) carbrayl; (4) atrazine; (5) methidathion; (6) azoxystrobin; and (7) propazine. Figure S5: typical chromatograms of blank (A), unspiked (B), and spiked (C) RSM milk sample concentration level 2 (100 *μ*g/L for CAR; 130 *μ*g/L for THE, CYZ, ATZ, and PRZ; 260 *μ*g/L for AZO; and 390 *μ*g/L for MET). Extraction conditions: sample size, 5 mL; extraction solvent, acetonitrile; extraction solvent volume, 1000 *μ*L; salt type, MgSO_4_; amount of MgSO_4_ added, 40% (m/v); extraction time, 0.5 min; pH of solution, 8.0; and centrifugation speed, 4000 rpm for 5 min. Peaks identifications: (1) thiamethoxam; (2) cyanazine; (3) carbrayl; (4) atrazine; (5) methidathion; (6) azoxystrobin; (7) propazine.
