# Supplementary material for: A Highly Selective Analytical Method Based on Salt-Assisted Liquid-Liquid Extraction for Trace-Level Enrichment of Multiclass Pesticide Residues in Cow Milk for Quantitative Liquid Chromatographic Analysis
Source: Int J Anal Chem. 2023 Sep 29;2023:1754956. doi: 10.1155/2023/1754956 (PMC10558272; doi:10.1155/2023/1754956)
Supplement: Supplementary Materials — Figure S1: effect of centrifugation time. Extraction conditions: sample size, 5 mL; extraction solvent, acetonitrile; extraction solvent volume, 1000 μL; salt type, MgSO4; amount of MgSO4 added, 40% (m/v); extraction time, 0.5 min; pH of solution, 8.0; and centrifugation speed, 4000 rpm; n = 6. Figure S2: calibration curve of (a) thiamethoxam, (b) cyanazine, (c) carbrayl, (d) atrazine, (e) methidathion, (f) azoxystrobin, and (g) propazine. Figure S3: chromatograms of individual pesticides: (1) thiamethoxam; (2) cyanazine; (3) carbrayl; (4) atrazine; (5) methidathion; (6) azoxystrobin; and (7) propazine. Figure S4: typical chromatograms of blank (A), unspiked (B), and spiked (C) PMM milk sample at concentration level 2 (100 μg/L for CAR; 130 μg/L for THE, CYZ, ATZ, and PRZ; 260 μg/L for AZO; and 390 μg/L for MET). Extraction conditions: sample size, 5 mL; extraction solvent, acetonitrile; extraction solvent volume, 1000 μL; salt type, MgSO4; amount of MgSO4 added, 40% (m/v); extraction time, 0.5 min; pH of solution, 8.0; and centrifugation speed, 4000 rpm for 5 min. Peaks identifications: (1) thiamethoxam; (2) cyanazine; (3) carbrayl; (4) atrazine; (5) methidathion; (6) azoxystrobin; and (7) propazine. Figure S5: typical chromatograms of blank (A), unspiked (B), and spiked (C) RSM milk sample concentration level 2 (100 μg/L for CAR; 130 μg/L for THE, CYZ, ATZ, and PRZ; 260 μg/L for AZO; and 390 μg/L for MET). Extraction conditions: sample size, 5 mL; extraction solvent, acetonitrile; extraction solvent volume, 1000 μL; salt type, MgSO4; amount of MgSO4 added, 40% (m/v); extraction time, 0.5 min; pH of solution, 8.0; and centrifugation speed, 4000 rpm for 5 min. Peaks identifications: (1) thiamethoxam; (2) cyanazine; (3) carbrayl; (4) atrazine; (5) methidathion; (6) azoxystrobin; (7) propazine. [file 1754956.f1.docx]

**Supplementary material**

**A Highly Selective Analytical Method Based on Salt Assisted Liquid-Liquid Extraction for Trace Level Enrichment of Multiclass Pesticide Residues in Cow Milk for Quantitative Liquid Chromatographic Analysis**

Habtamu Bekele^1^, Weldegebrie^l^ Yohannes^1^, Negussie Megersa^1*^

^1^Department of Chemistry, College of Natural and Computational Sciences, Addis Ababa University,

P. O. Box 1176, Addis Ababa, Ethiopia

****Corresponding Author:***

Prof. Negussie Megersa

Email: negussie.megersa@gmail.com

P. O. Box (Personal): 34706, Addis Ababa, Ethiopia

Telephone (mobile): +251-911-68-42-60

Fax: +251-111-23-94-70

***Co-authors:***

1. Habtamu Bekele

Email: bekelehabtamu16@gmail.com

2. Weldegebriel Yohannes

Email: wyohannes2001@gmail.com

S_1_

FIGURE S_1_: Effect of centrifugation time. Extraction conditions: sample size, 5 mL; extraction solvent, acetonitrile; extraction solvent volume; 1000 µL; salt type, MgSO_4_; amount of MgSO_4_ added, 40% (m/v); extraction time, 0.5 min; pH of solution, 8.0; centrifugation speed, 4000 rpm, n=6.

S_2_

FIGURE S_2_**:** Calibration curve of a) thiamethoxam, b) cyanazine, c) carbrayl, d) atrazine, e) methidathion, f) azoxystrobin,

g) propazine.

S_3_


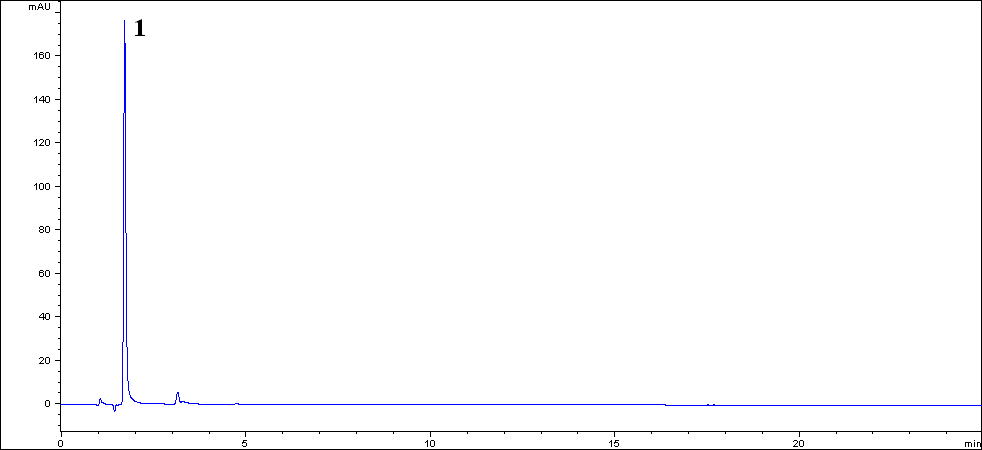

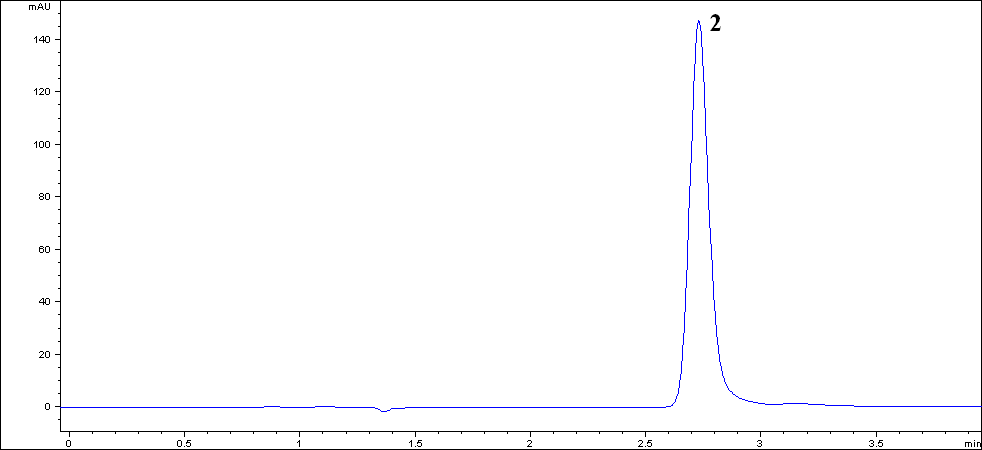


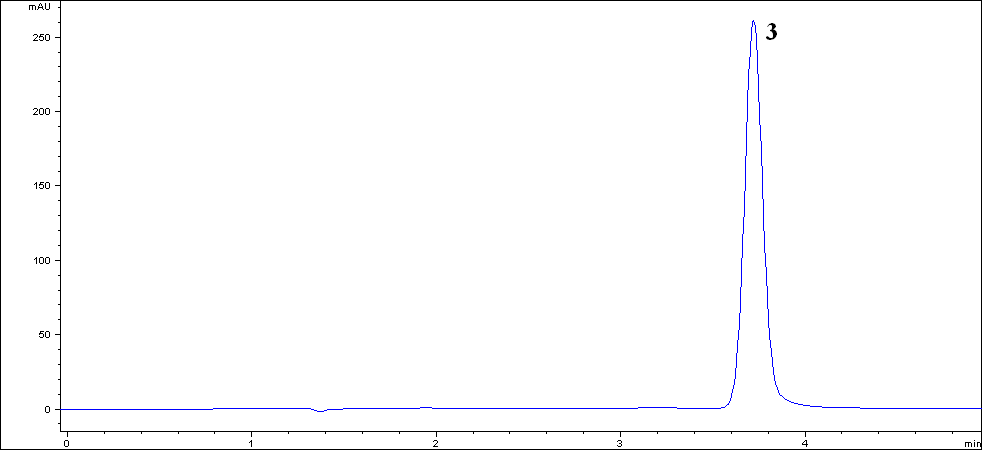

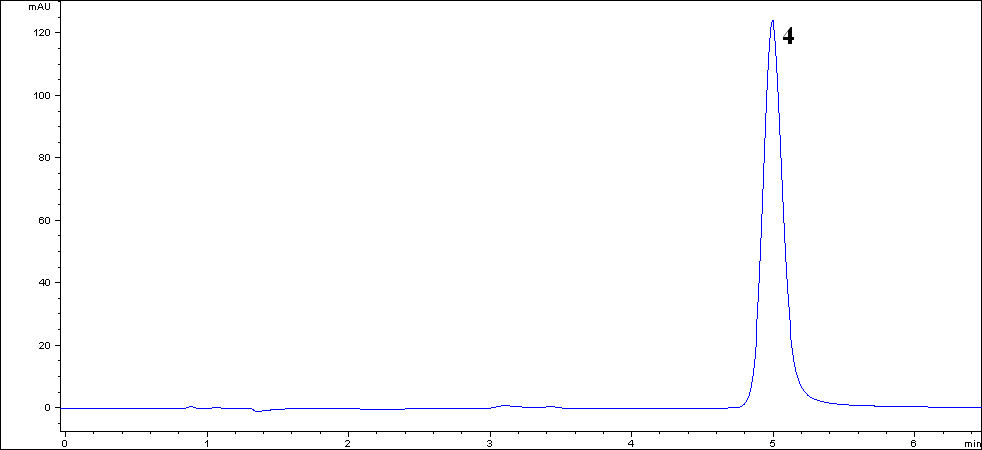


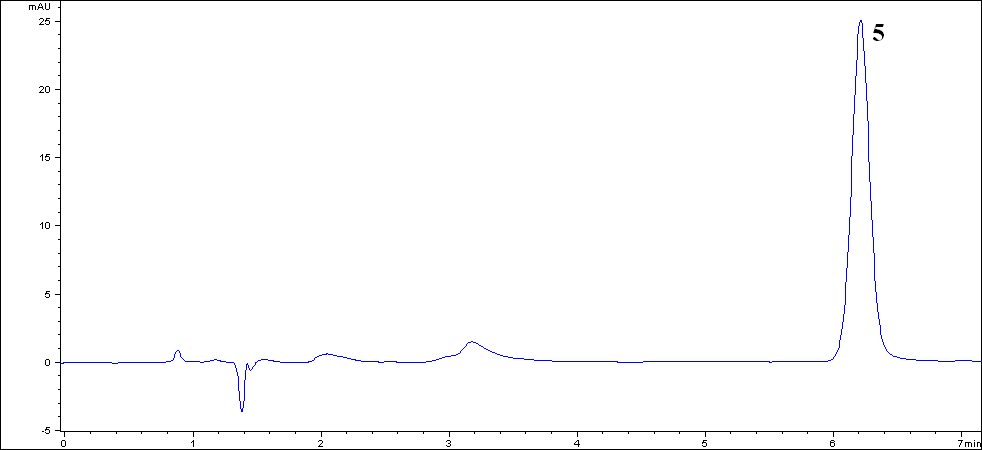

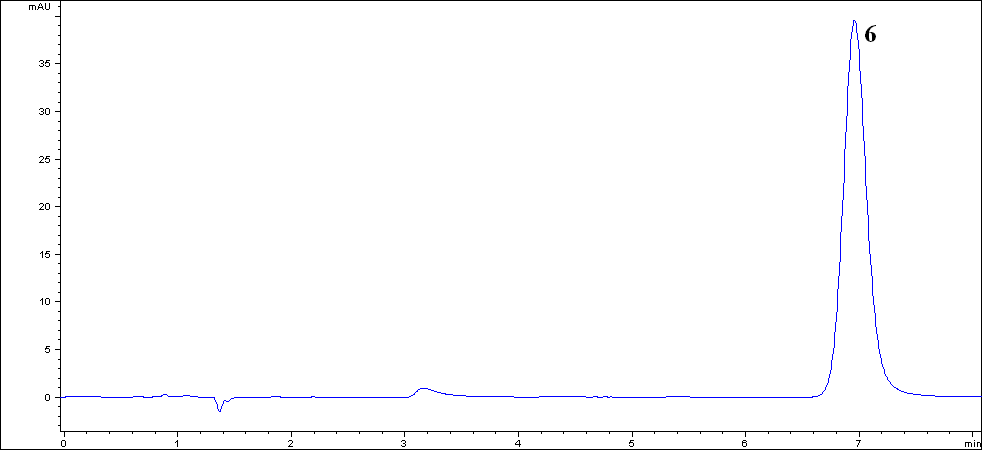


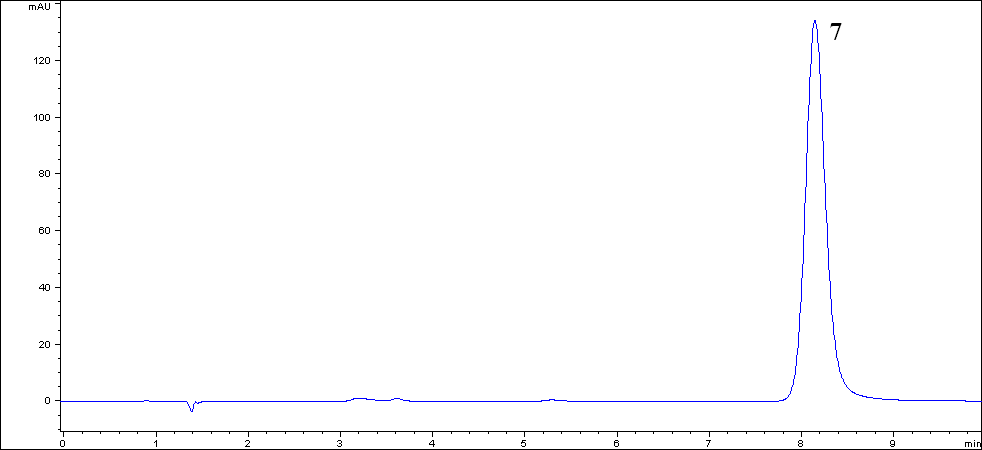


FIGURE S_3_**:** Chromatograms of individual pesticides: 1, thiamethoxam 2, cyanazine 3, carbrayl 4, atrazine 5, metadiothate 6, azoxystrobin 7, propazine.

S_4_


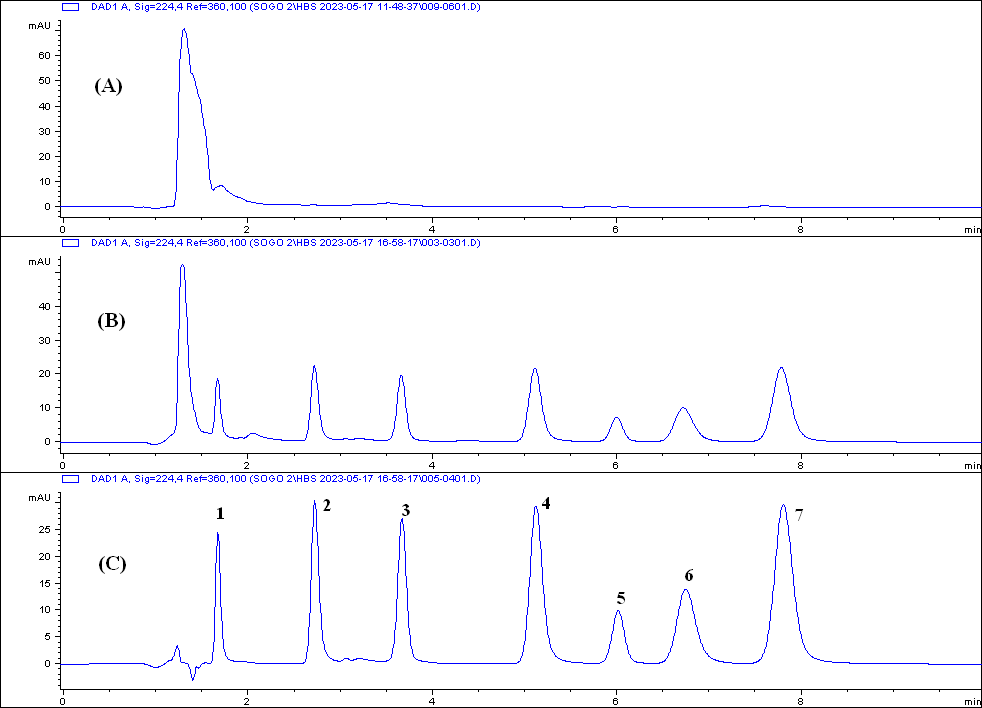


FIGURE S_4_**:** Typical chromatograms of blank (A), unspiked (B) and spiked (C) PMM milk sample at concentration level 2 (100 µg/L for CAR; 130 µg/L for THE, CYZ, ATZ, and PRZ; 260 µg/L for AZO; 390 µg/L for MET). Extraction conditions: sample size, 5 mL; extraction solvent, acetonitrile; extraction solvent volume; 1000 µL; salt type, MgSO_4_; amount of MgSO_4_ added, 40% (m/v); extraction time, 0.5 min; pH of solution, 8.0; centrifugation speed, 4000 rpm for 5 min. Peaks identifications: 1, thiamethoxam 2, cyanazine 3, carbrayl 4, atrazine 5, methidathion 6, azoxystrobin 7, propazine*.*


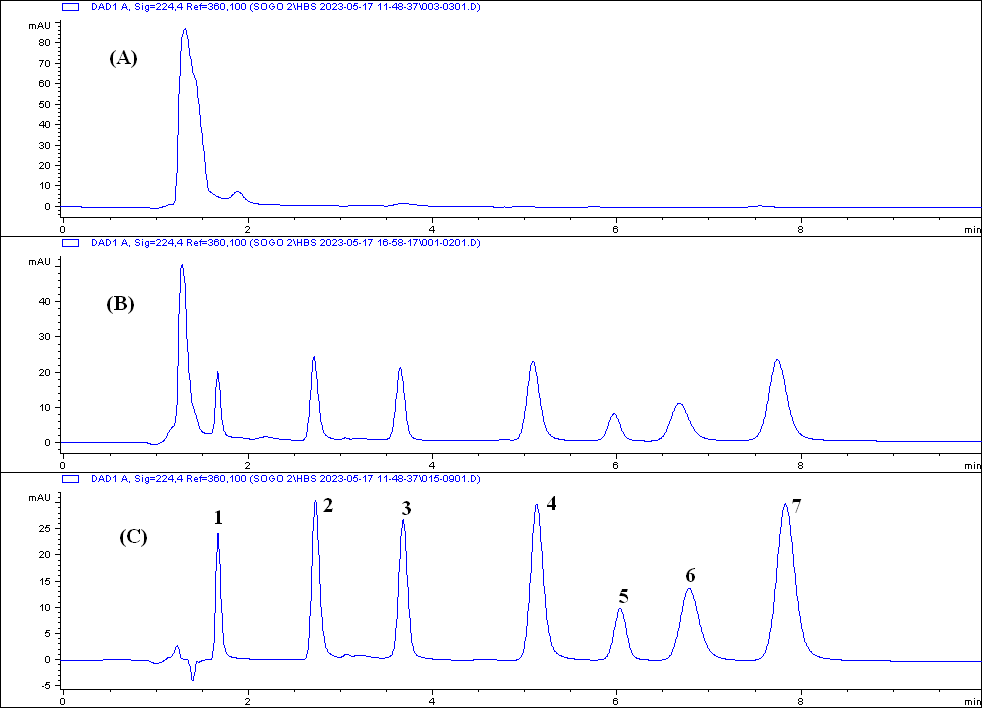


FIGURE S_5_**:** Typical chromatograms of blank (A), unspiked (B) and spiked (C) RSM milk at concentration level 2 (100 µg/L for CAR; 130 µg/L for THE, CYZ, ATZ, and PRZ; 260 µg/L for AZO; 390 µg/L for MET). Extraction conditions: sample size, 5 mL; extraction solvent, acetonitrile; extraction solvent volume; 1000 µL; salt type, MgSO_4_; amount of MgSO_4_ added, 40% (m/v); extraction time, 0.5 min; pH of solution, 8.0; centrifugation speed, 4000 rpm for 5 min. Peaks identifications: 1, thiamethoxam 2, cyanazine 3, carbrayl

4, atrazine 5, metadiothate 6, azoxystrobin 7, propazine.

S_5_
